# Mental Health Disparities Mediating Increased Risky Sexual Behavior in Sexual Minorities: A Twin Approach

**DOI:** 10.1007/s10508-020-01696-w

**Published:** 2020-04-19

**Authors:** Olakunle Ayokunmi Oginni, Patrick Jern, Frühling Vesta Rijsdijk

**Affiliations:** 1 The Social, Genetic and Developmental Psychiatry Centre, Institute of Psychiatry, Psychology and Neuroscience, King’s College London, Denmark Hill, London, SE5 8AF UK; 2 Department of Psychology, Åbo Akademi University, Åbo, Finland

**Keywords:** Sexual minority, Twin study, Mental health, Risky sexual behavior, Sexual orientation

## Abstract

Increased risky sexual behavior in sexual minorities relative to heterosexual individuals may be partly explained by mental health disparities, and both factors may be further jointly influenced by common genetic and environmental factors. However, these relationships have not been previously investigated. The objectives of the present study were to investigate mental health disparities as a mediator of the relationship between sexual orientation and risky sexual behavior, controlling for genetic and environmental effects in this relationship and testing for sex differences. Participants included 5814 twins from a Finnish twin cohort. Specified latent factors included sexual orientation, mental health indicators, and risky sexual behavior. Twin models were fitted to the factor structure of the data whereby a Cholesky decomposition on the factors was compared to a mediation submodel using OpenMx. Sex differences were tested in the final model. Phenotypically, mental health disparities partially mediated the relationship between sexual orientation and increased risky sexual behavior, with comparable effects in males and females. However, while this indirect route from sexual orientation to risky sexual behavior mainly contained transmitted genetic effects in males, there was a significant proportion of transmitted shared environmental effects in females. This is the first study to demonstrate that the mediation relationships between sexual orientation, mental health disparities, and risky sexual behavior are not confounded by genetic and environmental factors. The significant sex differences need to be recognized in future research and intervention design to improve sexual health in sexual minorities.

**Electronic supplementary material:**

The online version of this article (10.1007/s10508-020-01696-w) contains supplementary material, which is available to authorized users.

## Introduction

Since the recognition of the HIV epidemic over 35 years ago, sexual minority men have been disproportionately affected relative to heterosexual men, and this has continued till now (Beyrer et al., 2012). Risky sexual behaviors, which are more prevalent among sexual minority relative to heterosexual men (Glick et al., 2012), represent an important mechanism for this persistent disparity. These behaviors, which are independently associated with an increased likelihood of adverse sexual outcomes, include unprotected anal sex, multiple sexual partners, early age at sexual debut, and sex acts while under the influence of psychoactive substances (Beyrer et al., 2012). Similarly, the rates of risky sexual behavior and adverse sexual health outcomes, including unplanned pregnancies, are higher among sexual minority relative to heterosexual women (Tornello, Riskind, & Patterson, 2014). These increased rates were explained as a possible consequence of sexual minority women being more likely to be living on their own earlier compared to heterosexual women. This was either because they had been ejected from home or had left home to escape abuse or be more independent; however, it may also increase the risk for sexual victimization and engaging in other risky behaviors. While the significance of some indices of risky sexual behaviors may differ in sexual minority men and women (e.g., condomless anal sex), when indices common to males and females are investigated, the disparities between sexual minority and heterosexual individuals appear to be comparable across sex. For example, the ages at sexual debut in heterosexual men and women were 17.4 and 17.8 years, respectively, while the corresponding ages in sexual minority men and women were 15.4 (Glick et al., 2012) and 14.4–14.9 years, respectively (Tornello et al., 2014). Similarly, while heterosexual men and women reported a median of one sexual partner in the past 12 months, sexual minority men and women, respectively, reported a median of 2–4 (Glick et al., 2012) and 2.7–3.5 partners (Tornello et al., 2014) within the same time frame.

Sexual minority individuals also report higher rates of mental health problems relative to heterosexual individuals (King et al., 2008; Plöderl & Tremblay, 2015). These include depressive, anxiety, alcohol, and substance use disorders. Sex differences have also been reported in these disparities whereby the disparities for externalizing disorders (e.g., alcohol and substance use) are higher in sexual minority women (King et al., 2008; Plöderl & Tremblay, 2015). In contrast, the disparities for internalizing (e.g., depressive and anxiety) disorders are higher in sexual minority men (Plöderl & Tremblay, 2015).

Mental health problems, such as depressive and anxiety symptoms and problematic use of alcohol and other substances, are associated with increased risky sexual behavior. These relationships have been demonstrated in both the general population (Ramrakha, Caspi, Dickson, Moffitt, & Paul, 2000) and among sexual minority men (Halkitis et al., 2013) and women (Matthews et al., 2013). These findings partly explain elevated rates of risky sexual behavior within samples of sexual minorities; however, they do not explain the disparities in risky sexual behavior when sexual minority are compared with heterosexual populations. The higher rates of mental health problems among sexual minorities (Frisell, Lichtenstein, Rahman, & Långström, 2010) suggest the possibility that mental health disparities partly explain the observed risky sexual behavior disparities; however, few studies have investigated this relationship.

Findings from such cross-group comparisons will indicate the importance of eliminating mental health disparities in reducing disparities in risky sexual behavior. Sex differences in mental health disparities in general (Boyd et al., 2015) and sexual minority populations (Plöderl & Tremblay, 2015) further suggest the possibility of sex differences in this proposed relationship. For example, a longitudinal study showed that the positive relationship between risk for substance dependence disorder and number of sexual partners was significantly stronger among women compared to men in the general population (Ramrakha et al., 2013). In contrast, controlling for sex did not change the relationship between number of sexual partners and depressive and anxiety disorders. Considering that the disparity in substance use problems is higher in sexual minority women, it is possible that the relationship between mental health and risky sexual behavior disparities would be stronger in sexual minority women compared to men; however, this has not been investigated in the general population.

Based on the described relationships, we propose a mediation model whereby mental health disparities mediate the relationship between sexual orientation and increased risky sexual behavior. Considering that some of the indicators of later sexual minority status (e.g., childhood gender nonconformity; Li, Kung, & Hines, 2017) and the age at awareness of sexual orientation (11–19 years) usually temporally precede the onset of sexual activity (16–27 years) (Dunlap, 2016; Maguen, Floyd, Bakeman, & Armistead, 2002), it is pragmatic to consider sexual orientation as preceding risky sexual behaviors rather than vice versa. Similarly, analyses of longitudinal data indicate that sexual minority status is associated with higher rates of later mental health problems. For example, Oginni, Robinson, Jones, Rahman, and Rimes (2019) showed that despite adjusting for depressive symptoms at 12 years, adolescents who identified as non-heterosexual at 15 years still reported significantly higher rates of depressive symptoms at 18 years. Finally, findings from a longitudinal study in a general population provided stronger evidence for mental health problems being associated with later risky sexual behavior (Ramrakha et al., 2007) with a reduced likelihood of reverse causation.

The disparities in mental health and risky sexual behavior observed in sexual minority populations may be due to a common cause such as minority stress (Hatzenbuehler, 2009). This describes adverse experiences due to sexual minority status including discrimination, concealment of sexual orientation, expectation of stigmatization, and self-directed stigma. However, other evidence indicates that minority stress alone is an insufficient explanation for the mental health disparities among sexual minority persons. For example, rejection sensitivity (increased expectation and perception of discriminatory events and heightened emotional reactivity to these) may mediate or moderate the association between minority stress and mental health problems (Dyar, Feinstein, Eaton, & London, 2018; Feinstein, 2019). Furthermore, in a population-based study of Swedish twins, disparities in psychiatric diagnoses in non-heterosexual compared to heterosexual individuals were attenuated but not eliminated when minority stress factors were adjusted for (Frisell et al., 2010). This attenuation only became substantial or complete when familial factors were further adjusted for, though these factors could not be further resolved into additive genetic and/or common environmental effects.

This finding raises the possibility that common genetic and environmental factors may additionally influence the relationship between sexual orientation, and disparities in mental health and risky sexual behavior. This is further supported by evidence of genetic correlations between sexual orientation and depression (Ganna et al., 2019, Zietsch et al., 2012) and multiple sexual partners (Burri, Spector, & Rahman, 2015) with genetic factors explaining 60% and 78% of the phenotypic correlation of sexual orientation with depressive symptoms (*r* = .26; Zietsch et al., 2012) and with number of sexual partners (*r* = .13; Burri et al., 2015), respectively. Other support involves the genetic overlap between neuroticism, which is highly correlated with depression, and risky sexual behavior (*r* = .09, genetic correlation = .21; Zietsch, Verweij, Bailey, Wright, & Martin, 2010). However, no studies have investigated the potential influence of genetic and environmental factors on the proposed mediation relationship and whether sex differences in this relationship exist. Considering the different social influences on sexual behavior in men and women (Zietsch et al., 2010), it is possible that shared and/or non-shared environment influences will affect these relationships differentially in males and females.

In summary, several studies have investigated disparities in mental and sexual health outcomes among sexual minority compared to heterosexual individuals. Similarly, other studies have reported positive associations between adverse mental health outcomes and risky sexual behavior in the general population and in sexual minority samples. However, no study has previously investigated the association between mental and sexual health disparities in sexual minority and heterosexual individuals in a population-based sample. Findings from such a population-based approach may justify the continued need for social interventions to reduce mental health disparities and secondarily sexual health disparities. Controlling for confounding by variance component influences may further indicate potential targets for interventions. For example, persisting phenotypic associations after controlling for genetic and environmental confounding will indicate the need to target phenotypic rather than etiological associations. In contrast, significant confounding may indicate the need for research to further identify specific common etiological influences. Furthermore, significant sex differences may indicate different mechanisms for these disparities in sexual minority men and women. The objectives of this study were therefore to use a population-based sample of twins to determine (1) whether mental health disparities mediate the relationship between sexual minority status and increased risky sexual behavior; (2) whether this mediation is confounded by genetic and environmental factors; and (3) sex differences in these relationships. Specifically, we hypothesized that mental health disparities will increase the disparities in risky sexual behavior in sexual minority individuals, that these phenotypic mediation relationships will be confounded by shared genetic and environmental variance components, and that there will be sex differences in these relationships.

## Method

### Participants

Participants were derived from the second sample of the Finnish Genetics of Sexuality and Aggression cohort and consisted of monozygotic and dizygotic twins identified from the government-based registry of all Finnish citizens. Data were collected from Finnish-speaking twin pairs who were residing in Finland and aged 18–33 years at the time of data collection. Siblings aged at least 18 years were also targeted; however, only twins were included in the current analyses. Cover letters were sent to 23,577 adults who met these criteria, of which 958 declined to participate. Questionnaires were sent to the remainder, of whom 10,524 (including 6,531 twin individuals) responded. This gave a response rate of 45% which is comparable to rates typically obtained from mail surveys (Guo, Kopec, Cibere, Li, & Goldsmith, 2016) and sexuality-related research (Barth et al., 2016; Elliott et al., 2015). Of the twin participants, 717 were excluded as follows: 574 individuals had responded to less than 80% of the items for any of the questionnaires, and 143 individuals had indeterminate zygosities. This resulted in a study sample comprising 5814 participants: 658 male and 1378 female monozygotic twins, 671 male and 1219 female dizygotic twins, and 1888 opposite-sex twins. For further information, see Johansson et al. (2013).

Zygosity was determined through two questions (Sarna, Kaprio, Sistonen, & Koskenvuo, 1978), and accuracy was determined by genotyping a subset of the sample to be 91% which is acceptable (Christiansen et al., 2003). The DNA-determined zygosity was used if there was a discrepancy between this and question-based zygosity in individuals who were genotyped.

Ethical approval was obtained from the Ethics Committee of the Department of Psychology, Abo Akademi University, Finland, and informed consent was obtained from all participants.

### Measures

#### Covariates

These included age, assessed using a single question, and sex, ascertained from the Central Population Registry.

#### Sexual Orientation

This was ascertained by two questions: (1) “How often have you on average felt interest toward a member of the same sex?” and (2) “If an attractive woman (man for male participants), whom you like, proposes sexual interaction to you, how probable is it that you could do it (if you decide activity and nobody would ever know)?” These were, respectively, scored on a seven-point Likert scale ranging from “Never” (0) to “Every day” (6) and a six-point Likert scale ranging from “Impossible” (1) to “Very likely” (6). Cronbach’s alpha in this study was .67. Eighty percent of the participants had never felt interest toward members of the same sex, and 69.3% indicated that it was very unlikely or impossible that they would engage in sexual interaction with same-sex partners (Table S1, Supplementary material).

#### Depressive and Anxiety Symptoms

These were assessed using the depression and anxiety subscales of the 18-item self-report Brief Symptom Inventory (Derogatis, 2001). Each subscale consists of six questions each scored on a five-point Likert scale ranging from “Not at all” (0) to “Extremely” (4). Cronbach’s alphas for both subscales in the present study were .84 and .85, respectively. The scores in each subscale were summed and used in subsequent analyses with higher scores indicating higher levels of symptoms.

#### Substance Use

This was assessed for alcohol and cigarette-smoking. Alcohol use was assessed using the ten-item Alcohol Use Disorders Identification Test (AUDIT) which identifies and rates the severity of alcohol-related problems (Babor, Higgins-Biddle, Saunders, Monteiro, & World Health Organization, 2001). The items were rated on a five-point Likert scale with scores ranging from 0 to 4. Cronbach’s alpha in this study was .84. Cigarette-smoking was assessed using the two-item Heaviness of Smoking Index (HSI) (Heatherton, Kozlowski, Frecker, Rickert, & Robinson, 1989). The questions elicit the number of cigarettes smoked per day and the time to the first cigarette in the day (reverse-scored). The respective responses range from “Not at all” (scored 0) to “More than 30” (4) and “Less than 6 min” (1) to “More than 60 min” (4). Cronbach’s alpha in this study was .92. The item responses for each scale were summed and used in subsequent analyses. Higher scores indicated higher likelihood of alcohol- and tobacco-related problems.

#### Risky Sexual Behavior

This was assessed using the behavior subscale of the seven-item Sociosexual Orientation Inventory (Banai & Pavela, 2015). This subscale comprises three questions about the number of sexual partners in the past year, planned number of sex partners in the next 5 years, and the number of one-time sexual partners. This is consistent with findings from an international review identifying multiple partners as an indicator of HIV risk (Slaymaker, 2004). The responses were scored on a nine-point Likert scale ranging from “0” (0) to “20 or more” (8) and totals used in subsequent analyses. Cronbach’s alpha was .68 in the current study.

### Latent Factors

Three latent factors were constructed to reduce measurement error and facilitate twin model fitting for mediation analyses. The first factor (sexual orientation) was estimated by the two questions assessing sexual orientation. The second factor (mental health indicators, the mediator) was estimated by depressive and anxiety symptoms, and AUDIT and HSI scores. The decision to create a single latent mental health indicators factor was based on evidence indicating that this approach provided a better fit to the data (Halkitis et al., 2013) when investigating the association between mental health problems and risky sexual behavior. This approach is also consistent with a single underlying latent risk factor for mental health problems (Caspi et al., 2014). The third factor was risky sexual behavior (the outcome variable) which loaded on risky sexual behavior scores.

### Statistical Analysis

#### Data Preparation and Summary Statistics

Expectation–maximization imputation was carried out using SPSS version 25 (IBM IBM Corp, 2017). This method was selected due to its compatibility with maximum likelihood estimation methods. The expectation–maximization algorithm uses maximum likelihood estimation to find the best estimates with which to replace the missing data. It does this by deriving an expected fit function for the imputation model in a first step; in a second maximization step, the algorithm iteratively replaces missing items with approximate values until the algorithm converges (Dong & Peng, 2013). Item-level missingness ranged from .00 to 6.23% (this is depicted in Table S4, Supplementary material). Preliminary analyses including data summary statistics, and tests for phenotypic sex differences via ordinal logistic regression or Wilcoxon’s rank-sum test were carried out using STATA (StataCorp, 2016). Age and sex were adjusted for by individually regressing each variable on both and using the residuals in subsequent twin SEM analyses using OpenMx (Boker et al., 2011). The residuals were log-transformed to normalize the distributions for compatibility with parametric methods.

Model fit was determined by evaluating the chi squared value, degrees of freedom, and associated *p*-values. Nested models were compared by similar likelihood ratio testing, while best fit of non-nested models were determined by the Akaike information criterion (AIC) and Bayesian information criterion (BIC) indices.

#### SEM on Twin Data Structure

##### Constrained Phenotypic Correlation Models for Measured Variables

Correlations for the measured variables were investigated using maximum likelihood estimation. We applied constraints whereby all correlations differed across sex, but within-person correlations were equal across birth order and zygosity. In accordance with the assumptions of genetic models, this was to facilitate the estimation of many correlations using a reduced set of statistics. Symmetric across-twin correlations for monozygotic same-sex and dizygotic same- and opposite-sex twin pairs were estimated and inspected to determine the best variance component model. Details of the multivariate genetic model fitting are included in Supplementary material.

##### Multivariate Genetic Model Fitting

The multivariate genetic model parses variance of multiple variables into additive (*A*) or dominance or nonadditive (*D*) genetic, shared environmental (*C*), and unique environmental (*E*) components. This is done by comparing cross-twin within-trait correlations between monozygotic and dizygotic twin pairs. The method assumes that monozygotic and dizygotic twin pairs raised together share their common environment to the same extent, that monozygotic twin pairs are genetically identical, while dizygotic twin pairs have a correlation of .50 and .25 for *A* and *D* components, respectively, and that the unique environment is not correlated across both monozygotic and dizygotic twins (Rijsdijk & Sham, 2002). Both *D* and *C* components confound each other and are not simultaneously estimated. An *ACE* or *ADE* model is specified when the ratio of cross-twin within-trait correlations in monozygotic to dizygotic twins is less than or greater than 2, respectively. The ratios were generally less than two in our analyses, so the *ACE* model was specified.

A special correlated factor specification was fitted rather than the Cholesky decomposition to facilitate testing for qualitative sex differences (Neale, Røysamb, & Jacobson, 2006). These were determined by comparing the effects on model fit when *C* and *A* correlations across opposite-sex twins were alternately and simultaneously constrained to 1 and .5, respectively. Quantitative sex differences were investigated by observing the changes in model fit when the male and female loadings of the variance components on the variables were constrained to be equal, while keeping *A* and *C* correlations in opposite-sex twins fixed to .5 and 1, respectively (Neale et al., 2006). Based on finding significant quantitative sex differences, all subsequent models were tested for sex differences. Model fit was determined by evaluating the chi squared value, degrees of freedom, and associated *p*-values. Nested models were compared by similar likelihood ratio testing, while best fit of non-nested models were determined by the AIC and BIC indices (Berkout, Gross, & Young, 2014).

##### Phenotypic Factor Mediation Model with Factor Correlations by Zygosity

Phenotypic mediation was initially assessed in a model which specified three latent factors and three causal^1^ paths (sexual orientation → mental health indicators, mental health indicators → risky sexual behavior and sexual orientation → risky sexual behavior). The indirect effect was derived as the product of the standardized coefficients for paths sexual orientation → mental health indicators and mental health indicators → risky sexual behavior, while the total effect was derived as the sum of the indirect and direct (path sexual orientation → risky sexual behavior) effects (Baron & Kenny, 1986). The proportion of effect mediated was determined as the proportion of the indirect to the total effect. Factor correlations for the twin structure were estimated with constraints applied as in the correlation models for the measured variables.

##### Genetic Factor Mediation Models

To examine the etiological influences on the latent factors, we first specified a model in which the variance–covariance structure of the latent factors was specified as an *ACE* Cholesky decomposition (Genetic model 1). In addition, variable-specific variance components (*A*s, *C*s, and *E*s) were specified to load on each of the seven indicator variables. Next, a common factor *ACE* model (Genetic model 2) was specified to include variable-specific components. This model tested whether the factor covariances could be explained solely by one set of common components. In the next model (Genetic model 3), causal paths, factor-specific, and variable-specific *ACE* components were specified to test whether this was a more parsimonious description of the factor covariances.^2^ A better fit of the second model would indicate that the covariances of the three latent factors were more consistent with shared effects of common *A*, *C*, and *E* factors (i.e., including pleiotropy), while a better fit of the third model would indicate a combination of causal effects and transmitted component effects through the causal paths giving a more parsimonious explanation of the factor relationships (Rosenström et al., 2019). Sex differences were tested in the final model by alternately constraining factor-specific component loadings and the causal path coefficients to be equal in male and female twins.

#### Measurement Invariance

To ensure that the same constructs were being compared in male and female twins and that the comparisons are valid, we investigated measurement invariance for both phenotypic and genetic mediation models. As previously recommended (Putnick & Bornstein, 2016; Vandenberg & Lance, 2000), we specifically investigated configural, metric, scalar, and residual invariance. Configural invariance is inferred when identical factor structures including fixed and free loadings can be specified in both groups of participants being compared. Metric, scalar, and residual invariance occur when factor loadings, intercepts, and residual variances can, respectively, be constrained to be equal in male and female participants. At least partial metric invariance is required for between-group comparisons to be considered valid (Vandenberg & Lance, 2000). To further determine the impact of any measurement non-invariance, Putnick and Bornstein (2016) recommend that a fully invariant model (invariant and non-invariant items are constrained to be equal in comparison groups) be compared to partially invariant model (constraints are imposed on only invariant items). The impact of partial invariance is held to have little impact on the results if the conclusions from both the fully and partially invariant models are substantively comparable.

## Results

### Descriptive Analyses

The mean ages of the male and female participants were 26.2 (± 4.78) and 26.1 (± 5.09) years, respectively. The median scores and interquartile ranges for the other variables are shown in Table 1. Interest and probability of same-sex relationships and depressive and anxiety symptoms were significantly higher among females, while alcohol use, cigarette-smoking, and risky sexual behavior scores were significantly higher among males.

**Table 1:** Descriptive statistics of the observed variables per sex-zygosity groups

	Age mean (SD)	SOI median (range)	SOP median (range)	Depression median (range)	Anxiety median (range)	Alcohol use median (range)	Smoking cigarettes median (range)	RSB median (range)
MZM	25.0 (4.00)	.0 (0–6)	1.0 (1–6)	3.0 (0–22)	2.0 (0–21)	8.0 (0–31)	.0 (0–7)	4.0 (0–24)
DZM	25.0 (3.93)	.0 (0–6)	1.0 (1–6)	3.0 (0–24)	2.0 (0–23)	8.0 (0–29)	.0 (0–8)	4.0 (0–24)
MZF	25.3 (3.99)	.0 (0–6)	2.0 (1–6)	4.0 (0–24)	2.0 (0–24)	5.0 (0–26)	.0 (0–7)	4.0 (0–24)
DZF	24.9 (4.09)	.0 (0–6)	2.0 (1–6)	4.0 (0–24)	3.0 (0–24)	5.0 (0–28)	.0 (0–8)	4.0 (0–24)
DZO	25.2 (3.97)	.0 (0–6)	2.0 (1–6)	4.0 (0–24)	2.0 (0–24)	6.0 (0–28)	.0 (0–8)	4.4 (0–24)
Total	25.0 (4.00)	.0 (0–6)	2.0 (1–6)	4.0 (0–24)	2.0 (0–23)	6.0 (0–28)	.0 (0–7)	4.0 (0–24)

MZM = monozygotic male twins, DZM = dizygotic male twins, MZF = monozygotic female twins, DZF = dizygotic female twins, DZO = dizygotic opposite-sex twins, SOI = sexual orientation interest in same-sex relationship, SOP = sexual orientation probability of same-sex relationship, RSB = risky sexual behavior, SD = standard deviation

### Phenotypic Correlations

There were significant, positive within-person correlations between all the measured variables, and between the latent factors in males and females (Table 2). The between-twin, within-trait correlations for the seven variables and three latent factors in monozygotic twins were greater, but for the most part less than twice those in dizygotic twins for males and females (Table 3), indicating the effect of shared environment (*C*). The between-twin within-trait correlations in dizygotic opposite-sex twins were not significantly different from those of dizygotic same-sex twins. This suggested the absence of qualitative sex differences in the effects of the variance components.

**Table 2:** Within-individual correlations for observed variables and latent factors

	Male							Female						
	SOI (1)	SOP (2)	Depressive symptoms (3)	Anxiety (4)	Alcohol (5)	Smoking (6)	RSB (7)	SOI (1)	SOP (2)	Depressive symptoms (3)	Anxiety (4)	Alcohol (5)	Smoking (6)	RSB (7)
*Measured variables*														
(1)	1.00							1.00						
(2)	.69 (.66, .71)	1.00						.54 (.51, .56)	1.00					
(3)	.12 (.08, .16)	.14 (.10, .17)	1.00					.16 (.13, .20)	.15 (.12, .18)	1.00				
(4)	.08 (.04, .12)	.11 (.07, .18)	.67 (.65, .69)	1.00				.18 (.14, .21)	.13 (.10, .16)	.65 (.63, .67)	1.00			
(5)	.06 (.02, .09)	.08 (.05, .14)	.10 (.07, .13)	.10 (.07, .14)	1.00			.12 (.09, .15)	.12 (.10, .15)	.13 (.10, .16)	.13 (.10, .16)	1.00		
(6)	.01 (− .02, .05)	.01 (− .02, .04)	.09 (.06, .13)	.12 (.08, .15)	.30 (.26, .34)	1.00		.05 (.02, .08)	.06 (.04, .09)	.09 (.06, .12)	.09 (.06, .12)	.35 (.32, .38)	1.00	
(7)	.12 (.09, .16)	.13 (.10, .16)	.06 (.02, .10)	.06 (.02, .09)	.50 (.47, .53)	.26 (.22, .30)	1.00	.16 (.13, .19)	.17 (.14, .19)	.12 (.09, .15)	.08 (.05, .11)	.52 (.49, .54)	.30 (.27, .33)	1.00
Latent factors	SO	MHI	RSB					SO	MHI	RSB				
SO	1.00							1.00						
MHI	.20 (.14, .25)	1.00						.29 (.24, .33)	1.00					
RSB	.20 (.16, .33)	.13 (.08, .23)	1.00					.45 (.30, .50)	.28 (.18, .34)	1.00				

SOI—sexual orientation interest in same-sex relationship, SOP—sexual orientation probability of same-sex relationship, RSB—risky sexual behavior

Latent factors: SO—sexual orientation, MHI—mental health Indicators, RSB—risky sexual behavior

**Table 3:** Cross-twin within-trait correlations per sex-zygosity groups with 95% confidence intervals

	SOI	SOP	Depression	Anxiety	Alcohol use	Smoking cigarettes	RSB	SO^a^	MHI^a^	RSB^a^
MZM	.63 (.54, .70)	.53 (.41, .62)	.45 (.34, .54)	.42 (.28, .53)	.65 (.58, .71)	.66 (.57, .72)	.56 (.47, .63)	.92 (.80, 1.00)	.70 (.57, .80)	.54 (.45, 1.00)
DZM	.21 (.03, .35)	.28 (.08, .43)	.15 (− .01, .30)	.07 (− .10, .23)	.46 (.34, .56)	.31 (.16, .44)	.32 (.18, .44)	.41 (.08, .64)	.20 (− .03, .41)	.28 (.14, .71)
MZF	.31 (.23, .39)	.52 (.46, .58)	.37 (.30, .44)	.44 (.38, .50)	.62 (.57, .66)	.60 (.55, .64)	.51 (.45, .56)	.67 (.59, .75)	.57 (.48, .64)	1.00 (1.00, 1.00)
DZF	.25 (.16, .33)	.24 (.13, .34)	.25 (.15, .34)	.26 (.16, .35)	.27 (.18, .36)	.29 (.20, .37)	.24 (.16, .32)	.36 (.23, .47)	.37 (.25, .48)	.49 (.19, .65)
DZO	.11 (.00, .22)	.03 (− .05, .12)	.08 (− .01, .18)	.06 (− .04, .15)	.12 (.03, .20)	.15 (.06, .23)	.06 (− .02, .15)	.14 (.00, .27)	.12 (.00, .24)	.13 (.01, .28)

MZM = monozygotic male twins, DZM = dizygotic male twins, MZF = monozygotic female twins, DZF = dizygotic female twins, DZO = dizygotic opposite-sex twins, SOI = sexual orientation interest in same-sex relationship, SOP = sexual orientation probability of same-sex relationship, RSB = risky sexual behavior

^a^Latent factors: SO—sexual orientation, MHI—mental health indicators, RSB—risky sexual behavior

### Multivariate *ACE* Model Fitting

Genetic and environmental factors contributed significantly to the variance of the seven measured variables. Heritability estimates for the measured variables were higher in females (ranging from .27 to .58) compared to males (.10–.43) (Table 4). There were no qualitative sex differences. Quantitative sex differences were significant (*χ*^2^[21] = 1824.77, *p* < .001) comprising larger shared environmental effects for all the variables in men compared to women, and larger heritability estimates for sexual orientation variables, depressive symptoms, and anxiety symptoms.

**Table 4:** Standardized variance components and 95% confidence intervals for observed variables per sex

Variable	SOI	SOP	Depression	Anxiety	Alcohol	Smoking cigarettes	RSB	SO^a^	MHI^a^	RSB^a^
*h*^2^										
Male	.14 (.04, .26)	.10 (.02, .20)	.17 (.07, .27)	.17 (.08, .27)	.43 (.29, .54)	.42 (.29, .55)	.31 (.20, .42)	.60 (.29, .78)	.63 (.43, .76)	.32 (.09, .48)
Female	.27 (.20, .34)	.42 (.35, .48)	.35 (.29, .41)	.41 (.34, .47)	.58 (.51, .63)	.57 (.50, .62)	.48 (.42, .54)	.57 (.34, .70)	.29 (.05, .50)	.44 (.30, .54)
*c*^2^										
Male	.34 (.24, .44)	.52 (.42, .62)	.23 (.14, .32)	.19 (.12, .28)	.20 (.10, .32)	.16 (.05, .27)	.16 (.08, .27)	.10 (.00, .38)	.02 (.00, .18)	.18 (.05, .37)
Female	.02 (.00, .05)	.03 (.01, .06)	.02 (.00, .05)	.02 (.00, .05)	.08 (.04, .13)	.07 (.03, .12)	.05 (.02, .09)	.09 (.00, .28)	.21 (.04, .41)	.08 (.01, .19)
*e*^2^										
Male	.52 (.43, .62)	.37 (.30, .47)	.61 (.52, .70)	.64 (.54, .74)	.37 (.32, .44)	.42 (.34, .50)	.52 (.45, .60)	.29 (.19, .42)	.34 (.23, .48)	.50 (.42, .59)
Female	.71 (.64, .78)	.56 (.50, .62)	.63 (.58, .70)	.57 (.52, .63)	.35 (.30, .39)	.37 (.33, .42)	.47 (.42, .53)	.35 (.27, .43)	.50 (.42, .58)	.48 (.43, .54)

SOI—sexual orientation interest in same-sex relationship, SOP—sexual orientation probability of same-sex relationship, RSB—risky sexual behavior. *h*^2^, *c*^2^, *e*^2^: proportions contributed by additive genetic, shared and unique environmental factors, respectively

^a^Latent factors: SO—sexual orientation, MHI—mental health indicators, RSB—risky sexual behavior

### Phenotypic Factor Mediation Model

**Fig. 1:**
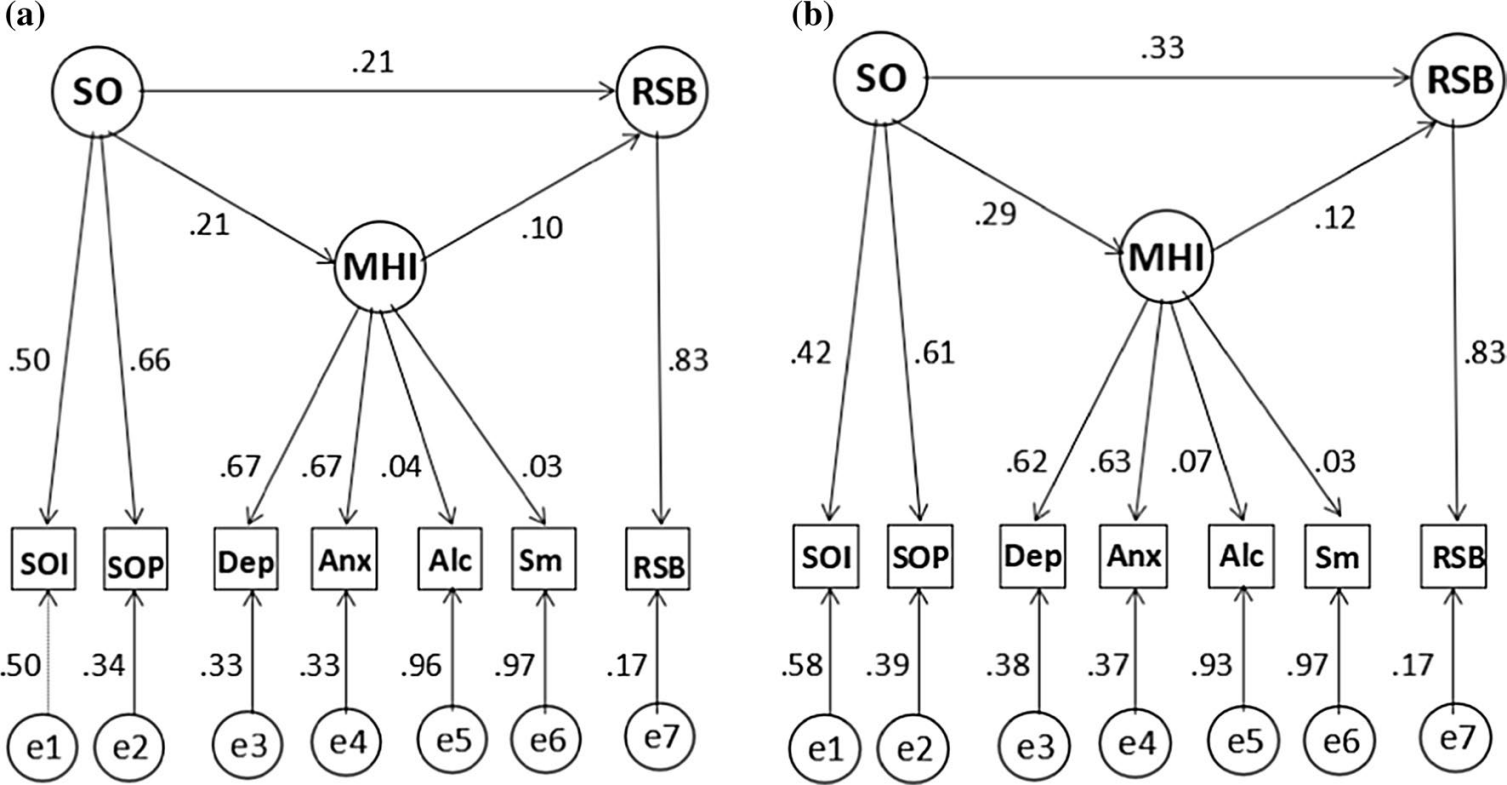
Phenotypic factor mediation model in males (**a**) and females (**b**): path diagrams depicting the mediation relationships between the factors: sexual orientation (SO), mental health indicators (MHI) and risky sexual behavior (RSB). Factor scales were estimated by fixing all first loadings to 1, and *e*1 and *e*2 were constrained to be equal for identification. Standardized path coefficients are reported. Indicator variables: SOI = sexual orientation interest in same-sex relationship, SOP = sexual orientation probability of same-sex relationship, Dep = depression, Anx = anxiety, Alc = alcohol use, Sm = smoking cigarettes, RSB = risky sexual behavior. *e*1–7—residual variance for the respective indicator variables

All the causal paths in the phenotypic analyses were statistically significant and comparable in males and females, indicating significant phenotypic mediation by the mental health indicators latent factor. The total effect of sexual orientation on risky sexual behavior was larger in women (.37; 95% CI .30–.50) but comparable to men (.23; 95% CI .16–.35) (Fig. 1). The direct and indirect effects of sexual orientation on risky sexual behavior were comparable and statistically significant in male (.21, 95% CI .14–.33 and .02, 95% CI .01–.04, respectively) and female twins (.33, 95% CI .26–.46) and .04, 95% CI .02–.06, respectively), as was the proportion of effect mediated by the mental health indicator factor (9% and 10%, respectively).

### Genetic Factor Mediation Model

**Fig. 2:**
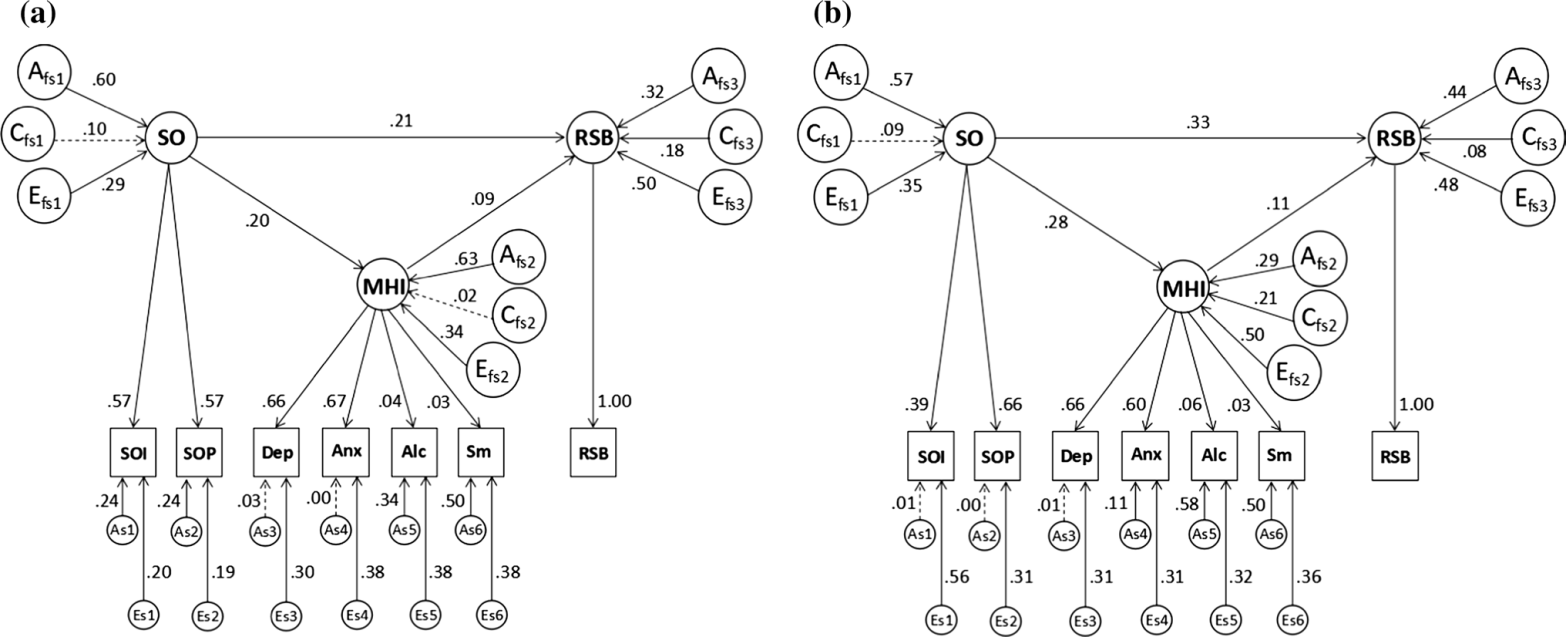
Genetic mediation model (Genetic model 3) in males (**a**) and females (**b**). This model included factor- and variable-specific variance components and causal paths. For identification, variable-specific paths were equated for SOI and SOP and fixed to 0 for the RSB indicator variable. *Note* Factors are SO—sexual orientation (indicators: SOI—sexual orientation interest in same-sex relationship, SOP—sexual orientation probability of same-sex relationship); MHI—mental health indicators (indicators: Dep—depression, Anx—anxiety, Alc—alcohol use, Sm—smoking cigarettes); RSB—risky sexual behavior (indicator: RSB—risky sexual behavior). Afs, Cfs, Efs—factor-specific additive genetic effects, shared environmental effects, unique environmental effects. Components for SO, MHI, and RSB indicators are indicated by numbers 1, 2, 3, 4, 5, and 6, respectively. As, Es—variable-specific additive genetic and unique environmental effects. Variable-specific C paths (Cs) were specified but not depicted here because only the loadings on alcohol (.26 in males) and smoking (.9 and .11 in males and females, respectively) were significant. These coefficients (and all 95% CIs) are, however, reported in Table S6 (Supplementary materials). All coefficients and factor loadings are standardized. Broken lines indicate nonsignificant paths

There was a significant worsening of fit in the second compared to the first genetic model (*χ*^2^[9] = 1490.75, *p* < .001). This indicated that the shared effects of the common *A*, *C*, and *E* factors alone were not sufficient to account for the covariance of the latent factors. The third genetic model in which factor- and variable-specific variance components and causal paths were specified (Fig. 2) was more parsimonious, relative to the other genetic models, shown by the lowest AIC and BIC values (Table S3, Supplementary material). There were significant *A* and *E* component loadings on each of the latent factors, and all the causal path coefficients remained statistically significant, similar to the phenotypic model. This means the covariance between the latent factors sexual orientation, mental health indicators, and risky sexual behavior can be best understood in terms of both the causal relationships and the transmitted influence of the *A* and *E* variance components.

Regarding the variance components loading on the latent factors in the final model, there were significant sex differences in the mental health indicators-risky sexual behavior path (*χ*^2^[9] = 195.20, *p *< .001). In males, this was significantly influenced by *A* and *E* components specific to mental health indicators which explained 63% and 33% of the covariance, respectively. In females, the influences of *A*, *C*, and *E* components specific to the mental health indicators latent factor were significant, explaining 41%, 16%, and 43% of the covariance, respectively. The other covariances (sexual orientation–mental health indicator and sexual orientation–risky sexual behavior) were comparably influenced by sexual orientation-specific *A* (60 and 57% in males and females, respectively) and *E* (29% and 35%) components. With respect to the causal path coefficients, there were significant sex differences (*χ*^2^[3] = 15.40, *p *= .002) when all three paths were tested simultaneously; however, individual path coefficients were not significantly different across sex.

### Measurement Invariance

Both the phenotypic and genetic mediation models in the present study demonstrated full configural invariance based on the identical factor structures and fixed and free loadings specified for male and female participants. We further demonstrated partial metric invariance as majority but not all factor loadings could be constrained to be equal in male and female participants. There was scalar non-invariance because the intercepts of the indicator variables could not be constrained to be equal in both groups, and there was partial residual invariance because some but not all the residual variances could be constrained to be equal in both groups of participants. These results are depicted in Table S5 (Supplementary material). Considering that we demonstrated at least partial metric invariance for our models, we can infer that the same conceptual framework was being compared in males and females (Vandenberg & Lance, 2000). We further explored the implications of partial measurement invariance by comparing fully invariant phenotypic and genetic mediation models with partially invariant models and found that the path estimates were comparable in both models. This indicated that the conclusions from both models were substantively comparable, i.e., partial non-invariance had little impact on the results.

## Discussion

This is the first analysis of a population-based twin cohort which shows that mental health disparities partially mediated the increased rates of risky sexual behavior among sexual minority compared to heterosexual individuals, within a genetically sensitive design. There were no significant sex differences in the direct and indirect phenotypic relationships between sexual orientation and risky sexual behavior. We further showed that these phenotypic relationships persisted after controlling for genetic and environmental influences and that shared environmental influences had a significantly larger role in the relationship between sexual orientation and risky sexual behavior in women.

While none of these direct and/or mediated relationships have been previously investigated using data from the present cohort, previous analyses of the present sample reported larger heritability estimates for alcohol use, smoking, and risky sexual behavior and no common environmental influences (von der Pahlen et al., 2008; Westerlund et al., 2010). This can be explained by our multivariate approach which increased the power to detect common environmental variance components (Schmitz, Cherny, & Fulker, 1998) which may previously have been incorporated into additive genetic influences. The heritability estimates for the mental health indicators and risky sexual behavior were, however, comparable with those from other studies (Broms, Silventoinen, Madden, Heath, & Kaprio, 2006; Jansson et al., 2004; Legrand, McGue, & Iacono, 1999; Mustanski, Viken, Kaprio, Winter, & Rose, 2007; Verhulst et al., 2018). The heritability estimates for sexual orientation indicators in female participants were comparable to those from previous studies (Bailey et al., 2016), but higher than those of male participants in the present study. This difference may be due to the better spread across the different categories of sexual orientation indicators in female compared to male participants in the present study (see Table S1, Supplementary materials), as has been previously reported (Oginni et al., 2019). This may have increased the power to estimate heritability in female relative to male participants. This sex difference was, however, attenuated in the heritability estimates for the sexual orientation latent factor and suggests that previous estimates may have been biased by measurement error.

In the first section of the indirect pathway, minority sexual orientation was significantly and comparably associated with increased mental health problems in men and women, and this has been linked to minority stress (Hatzenbuehler, 2009). Similar to prior findings (Frisell et al., 2010; Ganna et al., 2019; Zietsch et al., 2012), this relationship was further influenced by genetic and unique environmental factors. However, the influences of these factors were transmitted through phenotypic causal paths rather than as effects of common genetic and environmental factors. Prior explanations for this relationship include primary pleiotropic genetic influences on the hypothalamus–pituitary–gonadal (HPG) and adrenal (HPA) axes with secondary influences on sexual orientation and risk for mental health problems (Zietsch et al., 2012). Unique environmental factors such as in utero hormonal fluctuations and epigenetic factors may also jointly influence sexual orientation (Bailey et al., 2016; Rice, Friberg, & Gavrilets, 2013) and risk for later psychopathology (Hiroi, Carbone, Zuloaga, Bimonte-Nelson, & Handa, 2016; Radtke et al., 2015). While the above are possible explanations, our findings indicate that the genetic and environmental relationships between sexual orientation and increased mental health adversity are mediated by a phenotypic causal relationship rather than common etiological influences.

The second section of the indirect pathway indicated a positive phenotypic relationship between mental health indicators and risky sexual behavior. Phenotypically, this has been explained by cognitive and behavioral factors such as impaired safe-sex negotiation, regulation of negative affect with sex (Miltz et al., 2017), and impaired judgment and loss of control (Palamar, Acosta, Ompad, & Friedman, 2018). However, consistent with previous reports, there were further genetic and unique environmental influences on this relationship (Zietsch et al., 2010). Such genetic influences may indicate pleiotropic effects of an underlying vulnerability, while unique environmental factors may include extrafamilial stressful life events (Rijsdijk & Sham, 2002). However, again, our best-fitting model involved the transmission of genetic and environmental variance (risk) via a phenotypic causal path; that is, the etiological association between mental health indicators and risky sexual behavior is due to the phenotypic relationship between them. An important sex difference in this relationship was the larger effect of shared environmental effects in women. Although these influences are not specified in the twin design, a speculative candidate is early-life adversities which are associated with mental health adversities and risky sexual behavior in men and women (Hughes et al., 2017). This sex difference thus suggests a stronger influence of shared environmental factors on the relationship between mental health risky sexual behavior disparities in women compared to men; however, more aspects of the shared familial environment need to be investigated.

Despite the indirect effect of sexual orientation on increased risky sexual behavior, the largest proportion of this relationship was direct with no significant sex differences. The minority stress theory has been extended to explain this as a result of disruptions of self-regulatory mechanisms (Hatzenbuehler, 2009); however, few studies have demonstrated this effect for risky sexual behavior in sexual minority populations. Other explanations include sex activities at venues which facilitate risky sexual behavior such as clubs and bathhouses. Our finding of further genetic and non-shared environmental influences on this relationship is consistent with a previous finding (Burri et al., 2015), though this was only among women. We extend this by showing that these effects were comparable in men and women. Sexual orientation may be considered a component of a broader range of sexual behaviors (Bailey et al., 2016) including sexual drive which may determine the number of partners. Our findings show that the genetic relationship between sexual orientation and risky sexual behavior is most consistent with the transmission of genetic and environmental risk via a phenotypic causal path. Considering evidence that sexual orientation is determined prenatally (Bailey et al., 2016; Bogaert et al., 2018), non-shared environmental influences on this relationship may include aspects of the prenatal environment such gonadal hormone exposure. This has been shown to influence sexual behavior in animals (Henley, Nunez, & Clemens, 2011) and sexual orientation in humans (Bailey et al., 2016); however, its relationship with risky sexual behavior remains unclear.

Regarding the significance of our findings, phenotypic relationships between sexual orientation and mental health indices and risky sexual behavior suggest continuing effects of minority stress and the need for more efforts to reduce sexual orientation-based discrimination. These will reduce risky sexual behavior disparities by reducing mental health disparities in sexual minority relative to heterosexual individuals. The larger impact of shared environmental factors on the relationship between mental health indicators and risky sexual behavior among women suggests early-life adversities as risk indicators for adverse sexual health outcomes among sexual minority women with mental health problems. However, more research is needed to determine the impacts of early-life adversities and other shared environmental factors on this relationship. The mechanisms of the genetic relationships between minority sexual orientation, mental health, and risky sexual behavior also need to be further explored using other statistical and molecular genetic methods.

### Limitations

The strengths of this study include the use of a representative population-based sample and standardized instruments to assess the studied variables. However, in interpreting our findings, the following limitations must be considered.

Analyses were based on cross-sectional data, limiting the inference of causation; future studies should utilize longitudinal designs. The study sample was recruited from a developed country with higher acceptance for same-sex sexuality; thus, the findings may not generalize to less accepting regions; however, the mental health disparities in our study indicate that the sexual minority participants still experience significant minority stress. Our assessment of risky sexual behavior was based on the number of sexual partners which in and of itself may not be risky. However, our assessment is consistent with the association between multiple sexual partners and adverse sexual outcomes such as HIV and other sexually transmitted infections (Slaymaker, 2004; Tornello et al., 2014).

Violations of the underlying assumptions of the twin method such as the equal environments assumption and non-assortative mating of parents may mean that the estimates of genetic and environmental effects are biased. Similarities in trait correlations for twins reared apart and raised together indicate that the former assumption is negligible (Derks, Dolan, & Boomsma, 2006), while the latter can be investigated and adjusted for in future studies using extended twin designs (Rijsdijk & Sham, 2002).

## Electronic supplementary material

Below is the link to the electronic supplementary material.

Supplementary material 1 (DOCX 41 kb)

## Footnotes

1 We use the term “causal” to indicate phenotypic relationships as is commonly done in twin designs, to differentiate them from variance component loadings.

2 We use these terms to distinguish variable-specific variance components which load on the indicator variables and variance components which are specific to the latent factors.
