# Supplementary material for: Mental Health Disparities Mediating Increased Risky Sexual Behavior in Sexual Minorities: A Twin Approach
Source: Arch Sex Behav. 2020 Apr 19;49(7):2497–510. doi: 10.1007/s10508-020-01696-w (PMC7497451; doi:10.1007/s10508-020-01696-w)
Supplement: Supplementary file 1 — Supplementary material 1 (DOCX 41 kb) [file 10508_2020_1696_MOESM1_ESM.docx]

**Supplementary material**

**Table S1: Sex differences of the variables**

| **Variables** | **Male**  **n** | **%** | **Female**  **n** | **%** | **Total**  **n** | **%** | **Statistic** |
| --- | --- | --- | --- | --- | --- | --- | --- |
| **SOI** |  |  |  |  |  |  |  |
| **Never** | 1829 | 90.01 | 2843 | 75.17 | 4672 | 80.36 | 1.04***^a^ |
| **< Once a month** | 100 | 4.92 | 666 | 17.61 | 766 | 13.18 |  |
| **1-3 times/month** | 23 | 1.13 | 145 | 3.83 | 168 | 2.89 |  |
| **Once a week** | 13 | 0.64 | 42 | 1.11 | 55 | 0.95 |  |
| **2-3 times a week** | 15 | 0.74 | 26 | 0.69 | 41 | 0.71 |  |
| **4-6 times a week** | 10 | 0.49 | 14 | 0.37 | 24 | 0.41 |  |
| **Everyday** | 42 | 2.07 | 46 | 1.22 | 88 | 1.51 |  |
| **SOP** |  |  |  |  |  |  |  |
| **Impossible** | 1370 | 67.42 | 1295 | 34.24 | 2665 | 45.84 | 1.34***^a^ |
| **Very unlikely** | 365 | 17.96 | 1000 | 26.44 | 1365 | 23.48 |  |
| **Quite likely** | 79 | 3.89 | 390 | 10.31 | 469 | 8.07 |  |
| **Don’t know** | 66 | 3.25 | 359 | 9.49 | 425 | 7.31 |  |
| **Quite possible** | 80 | 3.94 | 509 | 13.46 | 589 | 10.13 |  |
| **Very likely** | 72 | 3.54 | 229 | 6.05 | 301 | 5.18 |  |
|  | **Median** | **IQR** | **Median** | **IQR** | **Median** | **IQR** |  |
| **Depression** | 3.0 | 5.00 | 4.0 | 5.00 | 4.0 | 5.00 | -7.95***^b^ |
| **Anxiety** | 2.0 | 3.00 | 3.0 | 4.00 | 2.0 | 4.00 | -9.58***^b^ |
| **Alcohol use** | 8.0 | 7.00 | 5.0 | 5.00 | 6.0 | 7.00 | 22.43***^b^ |
| **Smoking** | 0.0 | 2.00 | 0.0 | 2.00 | 0.0 | 2.00 | 5.82***^b^ |
| **RSB** | 4.2 | 7.00 | 4.0 | 5.00 | 4.0 | 6.00 | 4.64***^b^ |

Note: **SOI** - Sexual Orientation – Interest in same-sex relationship, **SOP** - Sexual Orientation - Probability of same-sex relationship, **RSB** = Risky sexual behaviour, **IQR** = Interquartile Range.

^a^Regression coefficient in which each variable was regressed on sex, ^b^Wilcoxon’s rank sum coefficient

****p* < 0.001

**Table S2a: Standardized variance component and factor loadings with 95% confidence intervals for the final genetic model in males.**

| **Component influence on factor variance-covariance** |  |  |  |  |  |  |  |  |  |
| --- | --- | --- | --- | --- | --- | --- | --- | --- | --- |
|  | **Afs** |  |  | **Cfs** |  |  | **Efs** |  |  |
|  | **SO** | **MHI** | **RSB** | **SO** | **MHI** | **RSB** | **SO** | **MHI** | **RSB** |
| **SO** | .60  (.29, .78) |  |  | .10  (.00, .38) |  |  | .29  (.19, .42) |  |  |
| **MHI** | .60  (.29, .78) | .63  (.43, .76) |  | .10  (.00, .38) | .02  (.00, .18) |  | .29  (.19, .42) | .34  (.23, .48) |  |
| **RSB** | .60  (.29, .78) | .63  (.46, .73) | .32  (.09, .48) | .10  (.00, .38) | .04  (.00, .18) | .18  (.05, .37) | .29  (.19, .42) | .33  (.24, .44) | .50  (.42, .59) |
|  |  |  |  |  |  |  |  |  |  |
| **Loadings on indicators** | **Latent Factors** | | | **Variable-specific component** | | | **Factor-specific component** | | |
|  | **SO** | **MHI** | **RSB** | **As** | **Cs** | **Es** | **Afs** | **Cfs** | **Efs** |
| **SOI** | .57  (.53, .60) |  |  | .24  (.16, .29) | .00  (.00, .06) | .20  (.15, .26) | .34  (.16, .45) | .06  (.00, .22) | .16  (.11, .24) |
| **SOP** | .57  (.54, .60) |  |  | .24  (.16, .29) | .00  (.00, .06) | .19  (.15, .23) | .34  (.17, .45) | .06  (.00, .22) | .17  (.11, .24) |
| **Dep** |  | .66  (.60, .73) |  | .03  (.00, .11) | .00  (.00, .09) | .30  (.23, .38) | .42  (.28, .52) | .01  (.00, .12) | .23  (.15, .32) |
| **Anx** |  | .67  (.61, .74) |  | .00  (.00, .06) | .00  (.00, .06) | .38  (.32, .46) | .43  (.29, .52) | .01  (.00, .12) | .23  (.15, .33) |
| **Alc** |  | .04  (.02, .05) |  | .34  (.12, .53) | .24  (.08, .43) | .38  (.32, .46) | .02  (.01, .04) | .00  (.00, .01) | .01  (.01, .02) |
| **Smok** |  | .03  (.02, .05) |  | .50  (.33, .61) | .08  (.01, .23) | .38  (.31, .47) | .02  (.01, .03) | .00  (.00, .01) | .01  (.01, .02) |
| **Rsb^b^** |  |  | 1.00 | .00  (.00, .00) | .00  (.00, .00) | .00  (.00, .00) | .32  (.09, .48) | .18  (.05, .37) | .50  (.42, .59) |
|  |  |  |  |  |  |  |  |  |  |

Notes: **Final Genetic Model** – Factor-specific variance components (**Asf, Csf, Esf**) are specified along with causal paths, while variable-specific variance components (**As,** **Cs, Es**) are specified in the bottom.

^a^The diagonals of each matrix give the proportion of factor variance determined by the respective variance component, while the off-diagonals represent the proportion of covariance determined.

^b^For identification, **RSB** loading on **Rsb** fixed to 1; **As**, **Cs** and **Es** loading of **Rsb** fixed to 0.

Latent factors: **SO** – Sexual Orientation (indicators: **SOI** – Sexual Orientation-

Interest in same-sex relationship, **SOP** – Sexual Orientation – Probability of same-sex relationship), **MHI** – Mental Health Indicators (indicators: **Dep** – Depressive symptoms, **Anx** – Anxiety, **Alc** – Alcohol, **Smok** – Smoking cigarettes), **RSB** – Risky Sexual Behaviour (indicator: **Rsb** – Risky Sexual Behaviour). **Afs, Cfs, Efs,** – Factor-specific Additive genetic effects, Shared environmental effects and Unique environmental effects, **As,** **Cs, Es** - Variable-specific Additive genetic effects, Shared environmental effects, and Unique environmental effects.

**Table S2b: Standardized variance component and factor loadings for the final genetic model with 95% confidence intervals in females.**

| **Component influence on factor variance-covariance** |  | |  |  |  |  |  |  |  |  |
| --- | --- | --- | --- | --- | --- | --- | --- | --- | --- | --- |
|  | **Afs** |  |  | **Cfs** |  |  | **Efs** |  |  |  |
|  | **SO** | **MHI** | **RSB** | **SO** | **MHI** | **RSB** | **SO** | **MHI** | **RSB** |  |
| **SO** | .57  (.34, .70) |  |  | .09  (.00, .28) |  |  | .35  (.27, .43) |  |  |  |
| **MHI** | .57  (.34, .70) | .29  (.05, .50) |  | .09  (.00, .28) | .21  (.04, .41) |  | .35  (.27, .43) | .50  (.42, .58) |  |  |
| **RSB** | .57  (.34, .70) | .41  (.23, .55) | .44  (.30, .54) | .09  (.00, .28) | .16  (.04, .30) | .08  (.01, .19) | .35  (.27, .43) | .43  (.37, .50) | .48  (.43, .54) |  |
|  |  |  |  |  |  |  |  |  |  |  |
| **Loadings on indicators** |  | | |  | | |  | | | |
|  | **Latent Factors** | | | **Variable-specific component** | | | **Factor-specific component** | | | |
|  | **SO** | **MHI** | **RSB** | **As** | **Cs** | **Es** | **Afs** | **Cfs** | **Efs** |  |
| **SOI** | .39  (.36, .42) |  |  | .01  (.00, .07) | .04  (.00, .08) | .56  (.51, .61) | .22  (.13, .28) | .03  (.00, .11) | .14  (.11, .17) |  |
| **SOP** | .66  (.63, .68) |  |  | .00  (.00, .04) | .02  (.00, .05) | .31  (.29, .34) | .37  (.23, .46) | .06  (.00, .18) | .23  (.18, .28) |  |
| **Dep** |  | .66  (.61, .71) |  | .01  (.00, .08) | .02  (.00, .06) | .31  (.26, .36) | .19  (.03, .33) | .14  (.02, .27) | .33  (.27, .39) |  |
| **Anx** |  | .60  (.55, .64) |  | .11  (.02, .16) | .00  (.00, .07) | .29  (25, .34) | .17  (.03, .30) | .13  (.02, .25) | .30  (.25, .35) |  |
| **Alc** |  | .06  (.04, .08) |  | .58  (.45, .65) | .04  (.00, .16) | .32  (.28, .36) | .02  (.00, .03) | .01  (.00, .03) | .03  (.02, .04) |  |
| **Smok** |  | .03  (.02, .04) |  | .50  (.34, .61) | .11  (.01, .25) | .36  (.32, .41) | .01  (.00, 02) | .01  (.00, .01) | .01  (.01, .02) |  |
| **Rsb^b^** |  |  | 1.00 | .00  (.00, .00) | .00  (.00, .00) | .00  (.00, .00) | .44  (.30, .54) | .08  (.01, 19) | .48  (.43, .54) |  |
|  |  |  |  |  |  |  |  |  |  |  |

Notes: **Final Genetic Model** – Factor-specific variance components (**Asf, Csf, Esf**) are specified along with causal paths, while variable-specific variance components (**As,** **Cs, Es**) are specified in the bottom.

^a^The diagonals of each matrix give the proportion of factor variance determined by the respective variance component, while the off-diagonals represent the proportion of covariance determined.

^b^For identification, **RSB** loading on **Rsb** fixed to 1; **As**, **Cs** and **Es** loading of **Rsb** fixed to 0.

Latent factors: **SO** – Sexual Orientation (indicators: **SOI** – Sexual Orientation-Interest in same-sex relationship, **SOP** – Sexual Orientation – Probability of same-sex relationship), **MHI** – Mental Health Indicators (indicators: **Dep** – Depressive symptoms, **Anx** – Anxiety, **Alc** – Alcohol, **Smok** – Smoking cigarettes), **RSB** – Risky Sexual Behaviour (indicator: **Rsb** – Risky Sexual Behaviour). **Afs, Cfs, Efs,** – Factor-specific Additive genetic effects, Shared environmental effects and Unique environmental effects, **As,** **Cs, Es** - Variable-specific Additive genetic effects, Shared environmental effects, and Unique environmental effects.

**Table S3: Model comparisons for genetic mediation models**

|  | **-2LL** | **df** | **AIC** | **BIC** | **Model compared with** | **Δ-2LL** | **Δdf** | ***p*-value** |
| --- | --- | --- | --- | --- | --- | --- | --- | --- |
| **Genetic Model 1** | 120943.01 | 40654 | 39635.01 | -216938.59 |  |  |  |  |
| **Genetic Model 2** | 122433.75 | 40633 | 41107.75 | -215522.64 | Genetic Model 1 | 1490.75 | 9 | <.001 |
| **Genetic Model 3** | 120950.84 | 40660 | 39630.84 | -216980.62 | Genetic Model 1 | 7.83 | 6 | 0.25 |
| **Genetic Model 4** | 119096.84 | 40622 | 37852.84 | -218518.79 |  |  |  |  |
| **Genetic Model 5** | 119292.04 | 40631 | 38030.04 | -218398.40 | Genetic Model 5 | 195.20 | 9 | <.001 |
| **Genetic Model 6** | 119112.25 | 40625 | 37862.25 | -218528.33 | Genetic Model 5 | 15.40 | 3 | 0.002 |

**Genetic Model 1:** Cholesky decomposition of variance components loading on latent factors, and variable-specific components loading on indicators. No causal paths were specified.

**Genetic Model 2**: Common-factor independent pathway model with common-factor components and variable-specific components specified. Factor-specific components causal paths were not specified.
**Genetic Model 3:** Final model - Submodel of Genetic Model 2 in which common components were dropped, factor-specific and variable-specific components and causal paths were specified.

**Genetic Model 4:** Sex differences model for Genetic Model 3.

**Genetic Model 5:** Homogeneity model for Genetic Model 4 in which factor-specific components were constrained to be equal in male and female twins.

**Genetic Model 6:** Homogeneity model for Genetic Model 4 in which causal paths were constrained to be equal in male and female twins.

**Table S4: Item-level missingness**

| **Variables** | **Number missing** | **% missingness** |
| --- | --- | --- |
| Age | 0 | 0.00 |
| Sex | 0 | 0.00 |
| Same-sex interest | 3 | 0.05 |
| Same-sex probability | 0 | 0.00 |
| BSI - Depression item 1 | 3 | 0.05 |
| BSI - Depression item 2 | 7 | 0.12 |
| BSI - Depression item 3 | 13 | 0.22 |
| BSI - Depression item 4 | 14 | 0.24 |
| BSI - Depression item 5 | 9 | 0.15 |
| BSI - Depression item 6 | 9 | 0.15 |
| BSI - Anxiety item 1 | 9 | 0.15 |
| BSI - Anxiety item 2 | 13 | 0.22 |
| BSI - Anxiety item 3 | 7 | 0.12 |
| BSI - Anxiety item 4 | 16 | 0.28 |
| BSI - Anxiety item 5 | 8 | 0.14 |
| BSI - Anxiety item 6 | 11 | 0.19 |
| AUDIT item 1 | 3 | 0.05 |
| AUDIT item 2 | 2 | 0.03 |
| AUDIT item 3 | 5 | 0.09 |
| AUDIT item 4 | 16 | 0.28 |
| AUDIT item 5 | 17 | 0.29 |
| AUDIT item 6 | 9 | 0.15 |
| AUDIT item 7 | 19 | 0.33 |
| AUDIT item 8 | 14 | 0.24 |
| AUDIT item 9 | 16 | 0.28 |
| AUDIT item 10 | 11 | 0.19 |
| HSI item 1 | 0 | 0.00 |
| HSI item 2 | 0 | 0.00 |
| RSB item 1 | 13 | 0.22 |
| RSB item 2 | 95 | 1.63 |
| RSB item 3 | 362 | 6.23 |

**BSI:** Brief Symptom Inventory; **AUDIT:** Alcohol Use Disorders Identification Test; **HSI:** Heaviness of Smoking Index; **RSB:** Risky Sexual Behavior.

**Table S5a: Tests of measurement invariance for the phenotypic model**

| **Model** | **χ2 (df)** | **Comparison model** | **Δχ2 (Δdf)** | **Decision** |
| --- | --- | --- | --- | --- |
| M1: Configural Invariance | 119994.00 (40635) | - | - | - |
| M2: Metric Invariance | 120013.10 (40639) | M1 | 19.07 (4)*** | Reject |
| M2a: Partial Metric Invariance | 119998.50 (40637) | M1 | 4.48 (2) | Accept |
| M3: Scalar Invariance | 120093.20 (40643) | M2a | 94.71 (6)*** | Reject |
| M3a: Partial scalar Invariance | 120003.60 (40638) | M2a | 5.12 (1)* | Reject |
| M4: Residual Invariance | 120864.50 (40642) | M2a | 865.98 (5)*** | Reject |
| M4a: Partial Residual Invariance | 119998.50 (40638) | M2a | 0.00 (1) | Accept |

**Table S5b: Tests of measurement invariance for the genetic model**

| **Model** | **χ2 (df)** | **Comparison model** | **Δχ2 (Δdf)** | **Decision** |
| --- | --- | --- | --- | --- |
| M1: Configural Invariance | 119096.80 (40622) | - | - | - |
| M2: Metric Invariance | 119265.30 (40626) | M1 | 168.41 (4)*** | Reject |
| M2a: Partial Metric Invariance | 119104.10 (40625) | M1 | 7.21 (3) | Accept |
| M3: Scalar Invariance | 119171.00 (40631) | M2a | 66.95 (6)*** | Reject |
| M3a: Partial scalar Invariance | 119109.00 (40626) | M2a | 4.99 (1)* | Reject |
| M4: Residual Invariance | 119333.60 (40630) | M2a | 229. 59 (5)*** | Reject |
| M4a: Partial Residual Invariance | 119107.80 (40620) | M2a | 3.71 (1) | Accept |

*** *p* < 0.05, ** *p* < 0. 01, *** *p* < 0.001**.
